# Atlanta Society of Medicine

**Published:** 1904-02

**Authors:** 


### ATLANTA SOCIETY OF MEDICINE.

THE following programme of the Atlanta Society of Medicine has been arranged for the year 1904:

January 7. Address by retiring President, Dr. L.P. Stephens; address by incoming President, Dr. A. W. Stirling.

January 21. The X-Ray in Medicine and Surgery. Dr. B. Wolff, Dr. M. B. Hutchins, Dr. J. N. Brawner, Dr. M. G. Campbell, Dr. E. H. Richardson, Dr. S. Barnett, Dr. M. Hoke.

February 4. *a*—Pneumonia, Dr. J. B. Baird. *b*—Mastoiditis; its causes, prevention and treatment, Dr. J. M. Crawford. Discussion: Dr. A. W. Calhoun, Dr. L. Crichton, Dr. F. H. Sims, Dr. W. E. Campbell.

February 18. *a*—Rheumatism—acute, Dr. J. N. Brawner. *b*—Rheumatism—chronic, Dr. C. D. Hurt. *c*—Rheumatism—Orthopedics and Rheumatic Joints, Dr. M. Hoke.

March 3. *a*—Imperfect Development of the Uterus, Dr. R. R. Kime. Discussion: Dr. V. O. Hardon, Dr. E. C. Davis. *b*—Chronic Poisoning, Dr. B. Wolff. Discussion: Dr. J. S. Todd, Dr. T. V. Hubbard, Dr. J. W. Duncan.

March 17. *A*—Epilepsy—*a*—Medical, Dr. S. G. C. Pinckney. *b*—Surgical, Dr. W. P. Nicolson. Discussion: Dr. E. Bates Block, Dr. J. N. Brawner. *B*—Displacements of the Uterus, Dr. S. Barnett. Discussion: Dr. V. O. Hardon, Dr. J. B. S. Holmes, Dr. R. R. Kime, Dr. J. G. Earnest, Dr. E. C. Davis, Dr. J. W. Duncan.

April 7. Headaches: *a*—From the Digestive Tract, Dr. J. N. LeConte. *b*—From the Eyes, Dr. W. E. Campbell. *c*—From the Nose, Dr. J. M. Crawford. *d*—From Other Causes, Dr. C. E. Boynton. Discussion: Dr. J. S. Todd, Dr. W. S. Elkin, Dr. J. B. Baird.

April 28. Syphilis—*a*—External, Dr. W. S. Goldsmith. *b*—Of Nervous System, Dr. S. G. C. Pinckney. *c*—Of Vascular System, Dr. C. D. Giddings. *d*—Of Other Organs, Dr. J. S. Todd. Discussion: Dr. W. B. Emery, Dr. W. L. Champion, Dr. M. B. Hutchins, Dr. B. Wolff, Dr. T. V. Hubbard.

May 5. Tubercle—*a*—Open Air Treatment of Tubercle of Lungs, Dr. J. L. Campbell. *b*—Tubercle of Larynx, Dr. F. H. Sims. *c*—Tubercle of Female Genito-Urinary Tract, Dr. E. G. Jones. *d*—Surgery of Tubercle, Dr. W. B. Armstrong. *e*—Pathology, Dr. Claud Smith. Discussion: Dr. C. G. Giddings, Dr. W. Westmoreland, Dr. F. W. McRae, Dr. W. P. Nicolson, Dr. G. H. Noble, Dr. V. O. Hardon, Dr. B. Wolff, Dr. M. B. Hutchins, Dr. J. N. Brawner.

May 19. *Parasites, Dr. H. F. Harris. Discussion: Dr. Claud Smith.

June 2. Albuminuria—*a*—So-called physiological albuminuria, also the connection between Albuminuria and disease, Dr. E. Bates Block. *b*—Effect on prognosis when existing before, and when appearing after, operations, Dr. H. P. Cooper. *c*—As effecting the eyes, Dr. A. W. Calhoun. *d*—In pregnancy, Dr. V. O. Hardon. *e*—In life assurance, Dr. W. E. Wilmerding. *f*—Pathology, Dr. Claud Smith. *g*—Operations for Bright's disease, Dr. W. P. Nicolson, Dr. F. W. McRae, Dr. E. C. Davis, Dr. R. R. Kime, Dr G. H. Noble.

*By Dr. Harris's invitation this meeting will be held at the College.

June 16. Gall Stones—*a*—Medical, Dr. Clarence Johnson. *b*—Surgical, Dr. C. Strickler. Discussion: Dr. F. W. McRae, Dr. W. S. Elkin, Dr. V. O. Hardon, Dr. S. Barnett, Dr. J. L. Campbell.

July 7. *a*—Extra-uterine pregnancy, Dr. E. C. Davis. Discussion: Dr. J. B. S. Holmes, Dr. G. H. Noble, Dr. R. R. Kime, Dr. J. G. Earnest, Dr. Dr. V. O. Hardon. *b*—Empyema, Dr. W. S. Elkin, Dr. Monroe Smith, Dr. S. Barnett, Dr. F. W. McRae.

July 21. Feeding and Diarrhoea of children: *a*—General considerations, Dr. L. P. Stephens. *b*—Auto-intoxications of childhood, Dr. Mariou Hull. *c*—The prevention of diarrhoea, with special reference to bacteriology, Dr. R. W. Hynds. Discussion: Dr. E. Van Goidtsnoven, Dr. C. E. Boynton, Dr. S. A. Visanska, Dr. C. E. Murphey, Dr. H. F. Harris.

August 4. *a*—Gastric cases, Dr. Clarence Johnson. Discussion: Dr. E. Bates Block, Dr. J. N. LeConte. *b*—Fibroid tumors of the uterus, Dr. J. G. Earnest, Dr. G. H. Noble, Dr. V. O. Hardon, Dr. R. R. Kime, Dr. J. B. S. Holmes, Dr. E. C. Davis.

August 18. Typhoid Fever. *a*—General considerations, Dr. T. V. Hubbard. *b*—In children, Dr. C. E. Boynton. *c*—Methods of prevention, including inoculation, Dr. J. P. Kennedy. Discussion: Dr. C. Strickler; Dr. W. C. Jarnagin, Dr. J. B. Baird. Dr. L. P. Stephens, Dr. L. B. Clark, Dr. L. Amster, Dr. W. S. Kendrick, Dr. S. Barnett.

September 1. The Tics, including chorea, Dr. E. Bates Block. Discussion: Dr. S. G. C. Pinckney, Dr. J. N. Brawner, Dr. W. S. Elkin, Dr. W. P. Nicolson, Dr. J. M. Crawford.

September 15. Gonorrhea. *a*—Including general effects. Dr. W. L. Champion. *b*—Urethral idiosyncrasies, Dr. W. B. Emery. *c*—Effects on women, Dr. V. O. Hardon. *d*—"Some genito-urinary points," Dr. L. Amster. Discussion: Dr. W. S. Goldsmith, Dr. E. C. Davis.

October 6. The Anemias, Dr. H. F. Harris. Discussion: Dr. J. S. Todd, Dr. E. Bates Block, Dr. J. L. Campbell.

October 20. *A*—Malignant Tumors. *a*—Operable *vs.* Inoperable, Dr. W. F. Westmoreland. *b*—Recent investigations in connection with etiology and pathology, and differential diagnosis from benign growth, Dr. Claud Smith. *c*—General remarks, Dr. J. McF. Gaston. *B*—The therapeutics of animal extracts, Dr. J. D. Cromer. Discussion: Dr. E. Bates Block, Dr. T. V. Hubbard, Dr. J. S. Todd.

November 3. *a*—Rabies, Dr. Claud Smith. *b*—The effects of various diets on health, Dr. R. R. Kime. Discussion: Dr. J. P. Kennedy, Dr. H. F. Harris, Dr. R. W. Hynds.

November 17. Alcohol. *a*—Physiological effects and food values, Dr.

R. W. Hynds. *b*—Effect of moderate drinking on prognosis in illness, Dr. J. S. Todd. *c*—As a drug during illness, with special reference to atheromatous arteries, Dr. W. S. Kendrick. *d*—The abuse of alcohol, W. B. Parks. *e*—Acute poisoning, Dr. C. C. Stockard. Discussion: Dr. R. R. Kime, Dr. S. G. C. Pinckney, Dr. W. S. Elkin, Dr. H. F. Harris.

December 1. Anesthetic, Dr. J. H. Johns, Dr. C. Strickler, Dr. W. L. Gilbert.

December 15. *a*—Annual message of the retiring President, Dr. A. W. Stirling. *b*—Election of officers.

